# Cutting-Edge Approaches in the Co-Amorphization Process

**DOI:** 10.3390/pharmaceutics17070850

**Published:** 2025-06-29

**Authors:** Azza A. K. Mahmoud, Géza Regdon, Katalin Kristó

**Affiliations:** Institute of Pharmaceutical Technology and Regulatory Affairs, University of Szeged, Eötvös u. 6., H-6720 Szeged, Hungary; khalid.azza.asim@o365.u-szeged.hu (A.A.K.M.); regdon.geza.laszlo@szte.hu (G.R.J.)

**Keywords:** co-former, amino acids, drug–drug co-amorphization, density functional theory, principal component analysis

## Abstract

**Background:** Recently, the co-amorphization method has been widely used to refine the bioavailability characteristics of poorly soluble drugs in addition to overcoming the drawbacks of other traditional amorphization techniques. **Objectives:** The main aim of this systematic review is to present an extensive outline of different co-former classes, co-former selection, and evaluation of produced co-amorphous systems. Methods: The systematic research was carried out based on three different databases, including PubMed, Scopus, and Web of Science time using co-amorphous, co-former, and drug as keywords. The selected papers were written in the English language and published between 2016 and 2024, and they focused on the co-amorphous systems, while articles discussing other amorphization techniques and crystallization processes were excluded. **Results:** 127 peer-reviewed articles were selected and summarized. **Conclusions:** This paper revealed that amino acid is the most commonly used co-former, specifically arginine with acidic drugs and tryptophan with acidic and basic drugs, and it reported other co-formers that were used and different co-amorphous systems with their dissolution behaviour and stabilities, and different computational tools that were applied in the selection of co-former and process result evaluation.

## 1. Introduction

The selection of physical drug form is one of the most critical parameters affecting drug therapeutic efficacy and is mainly related to the intended characteristics of the final dosage form. Although the use of crystal drug form in pharmaceutical industries ensures high physical and chemical stability of the dosage form in addition to monitoring of purification, it is not a suitable option for poorly soluble drugs (BCS II and IV), and not all drugs can be developed in crystal form. So, substituting the crystal form with an irregular configuration amorphous form is considered a suitable alternative that improves wettability, solubility, and bioavailability due to higher free energy and an undefined molecular arrangement. However, high molecular mobility of the amorphous form leads to recrystallization is considered a huge drawback that threatens stability, and it requires optimization and continuous control of environmental conditions during and after the amorphization process [1,2].

The amorphization process can be carried out by different techniques like solid dispersion, in situ amorphization, and co-amorphization. Although solid dispersion is currently applied in pharmaceutical research, it has low drug loading and a high risk of crystallization during preparation and storage due to polymer hygroscopicity and consequently phase transition from amorphous form to crystalline form. On the other hand, the low miscibility of the polymer makes it not suitable for small drug dosage [3,4].

Production of stable co-amorphous preparations is mainly based on using small-molecule co-formers that have high miscibility with the required drug, ensuring elevation of glass transition temperature (Tg). Moreover, it stabilizes amorphous drugs through the formation of strong molecular interactions, either strong ionic bonds or weak covalent bonds (π–π bond and hydrogen bond) [2,5,6].

Amino acid, organic acid, surfactant, and sugar were commonly used as co-formers for crystalline drugs. Also, drugs can be used as a co-former for the formulation of drug–drug co-amorphous systems by different methods that include ball milling, solvent evaporation, spray drying, quench melting, and freeze-drying [7].

Although co-amorphization techniques were mentioned in several studies, few of them emphasized herbal co-formers and modern tools used in process evaluation. This research aimed at providing a comprehensive description of recently used co-former types in the co-amorphization process, besides selection criteria and modern process evaluation during the last eight years.

## 2. Materials and Methods

Web of Science, PubMed, and Scopus databases were applied in systematic searching using (co-amorphous), (co-former), and (drug) as keywords. The selected research articles were published between 2016 and 2024 in the English language and related to pharmaceutical science.

The researches was limited to the co-amorphization of drugs using different co-formers, different selection methods, and preparation techniques, and research comparing between those techniques were analyzed and summarized while review articles, conference papers, and articles emphasized on other amorphization techniques like the solid dispersion method and mesoporous particles-based method were not considered and excluded.

## 3. Co-Former Types

The identification of the most suitable co-former (Table 1) is a critical step that ensures the production of the co-amorphous system with the required solubility, stability, and bioavailability.

**Figure 1:**
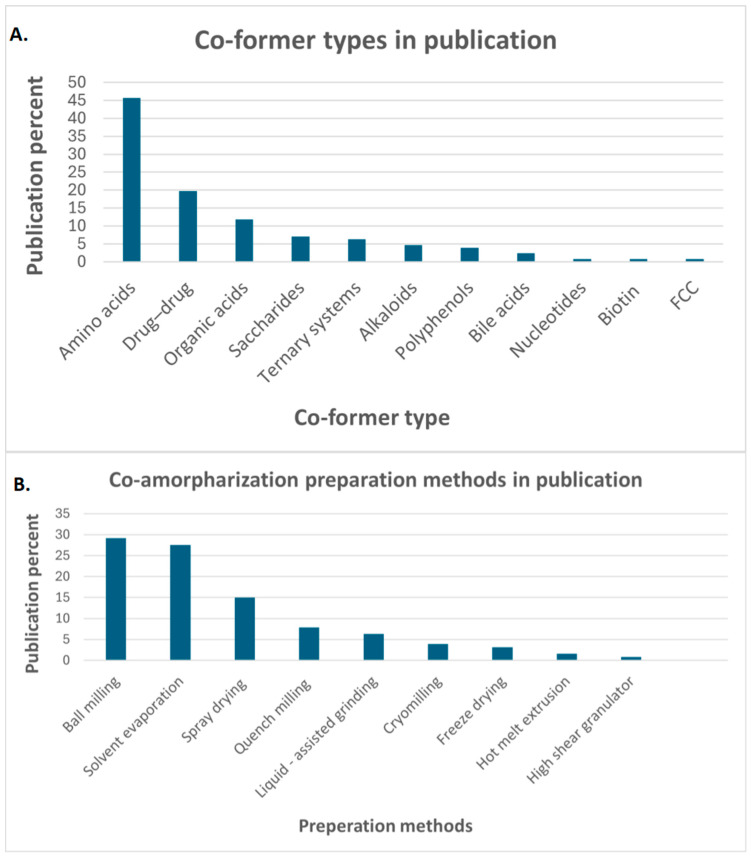
Statistical recording of (**A**). co-former types in publications, (**B**). co-amorphization preparation method in publications, functionalized calcium carbonate (FCC).

Different co-formers were utilized in the co-amorphization process in the last decade. Therefore, the most important articles related to co-formers were selected and statistically summarized, including their types and the applied preparation method (Figure 1).

### 3.1. Bile Acid

The use of bile acids has a significant effect on delaying crystallization by inhibiting crystal growth; in addition, it functions as a surfactant that improves the solubilization of drugs and micellar absorption. This effect is dependent on bile salt concentration and structure; this appeared in higher dissolution enhancement of the cholic acid pyrimethamine co-amorphous system has the highest AUC by a 25-fold increase of crystalline drugs, in addition to the highest dissolution rate. On the other hand, it enhanced lamotrigine AUC twofold, but it did not improve the dissolution rate of the trimethoprim co-amorphous system [8].

Moreover, applying cholic acid and cholic acid derivatives like ursodeoxy cholic acid with three model drugs (indomethacin, naproxen, and nifedipine) at molar ratios (1:0.5, 1:1, 1:2, 1:5) lead to the preparation of three amorphous form at molar ratio (1:2) and found nifedipine co-amorphous system has a higher solubility and stability than polyvinyl pyrrolidone (PVP) solid dispersion that makes it a good alternative to suspension preparation due to less water absorption [9].

Despite carboxylic acid and deoxycholic acid having no significant effect on the dissolution profile compared with crystalline sulfamerazine due to the accumulation of powder and poor dispersibility, sodium bile acid enhances solubility [4].

### 3.2. Organic Acid

The utilization of organic acids as co-formers in co-amorphization processes has been widely mentioned in previous research, as some have beneficial therapeutic effects. Tannic acid is a natural product containing polyphenolic groups, and it has anti-mutagenic and anti-oxidant effects, so it was used as a co-former with carbamazepine and indomethacin. According to X-ray powder diffraction (XRPD) results, a successful amorphous combination of the different molar concentrations of carbamazepine-tannic acid co-amorphous powder was formed. In contrast, indomethacin only (2:1) molar ratio formed an amorphous system due to the hydrogen bond between the carbonyl group and the two nitrogen atoms’ resonance. Additionally, it was found that molar ratios 1:1, 1:2, and 1:3 of indomethacin or carbamazepine with tannic acid co-amorphous have a higher solubility and intrinsic dissolution rate (IDR) than crystalline drugs (10-fold and 3-fold, respectively). Furthermore, all formulations of co-amorphous carbamazepine showed good stability for 6 months under different conditions (4, 25, 40 °C)/dry condition and 20 °C/60% RH; however, the indomethacin co-amorphous system was crystallized after one month and this may be related to lower interaction between indomethacin and co-former [10].

Additionally, the neuroprotective effect of shikimic acid encouraged researchers to use it as a co-former to the anti-psychotic drug (lurasidone) and it produced a faster dissolution rate at pH 2 and pH 3.8 at molar ratio 1:2 than other ratios (1:1 and 2:1) and crystalline drug but all formulations showed a hygroscopic behaviour that may due to large surface area of amorphous powder in addition to formation of hydrogen bonds [11].

Gallic acid is an organic acid that has anti-oxidant and anti-inflammatory effects that make it a good candidate for amorphized anti-cancer drugs. It enhanced the dissolution rate of erlotinib by 4.4-fold, besides improving maximum serum concentration (C_max_) by 7.9-fold and minimizing the time of peak plasma concentration (T_max_). However, it requires storage at low humidity conditions due to recrystallization at 75% RH [12].

Another phenolic acid co-former was used to obtain a sustained release co-amorphous system of sinomenine that includes salicylic acid, 2,3-dihydroxy benzoic acid, and 2,4-dihydroxy benzoic acid. All those co-formers improved the dissolution rate > 90% especially salicylic acid, which completely dissolved in 6 h with a higher dissolution percentage. Also, all preparations stayed stable for a long time (3 months at 25 °C/75% RH and for 12 months at low humidity conditions) [13].

The β-glucuronidase inhibition effect of chlorogenic acid has a significant role in the elimination of gelation and improvement of dissolution of curcumin and forming a stable co-amorphous system [14].

Three research studies are making comparisons between different organic acids. The first one compared the effect of different molar ratios of benzoic acid, citric acid, and malic acid on the amorphization of a basic model drug (carvedilol). It concluded that all formulations produce amorphous systems except the 1:4 ratio of benzoic acid co-former. Although molar concentration has a significant effect on the salt formation and the increase of Tg, malic acid and citric acid at a ratio of 1:3 do not have high Tg and salt formation due to steric hindrance. These observations agreed with stability results that proved increasing the content of organic acids has a negative effect on drug stability. Among all preparations 4:1 ratio has excellent stability for 6 months at 25 and 40 °C [15]. Also, Shi et al. compare different organic acids (tartaric acid, citric acid, succinic acid and malic acid) and found both molar concentrations 1:1 and 2:1 of tartaric acid have the highest solubility and dissolution rate (3.5–5.5 times and 4.3 times) than crystalline ibrutinib follow by citric acid and malic acid. According to stability test results of molar concentration 2:1 of tartaric acid and citric acid, the co-amorphous system remained stable for 180 days, while 1:1 molar concentration formed a cake at different time intervals [16]. Another study compared succinic acid and fumaric acid co-amorphous systems with co-crystal systems. Based on dissolution results, it was found that both organic acids exhibit a lower release percentage than the co-crystal system, especially fumaric acid, which forms sticky agglomerates with poor dispersibility and gelation behaviour [17].

In contrast, succinic acid was successful in improving the dissolution rate of palbociclib than other organic acids (tartaric acid, citric acid, malic acid). Also, both succinic acid and malic acid co-amorphous systems have good stability at 40 C°/75% RH for 3 months [18].

Citric acid is an aromatic agent for the preparation of oral dispersible tablets, and it has a beneficial effect in masking unpleasant tastes besides enhancing the dissolution properties of loratadine. Among the three molar ratios, 1:1 has the highest improvement of dissolution, about 3-fold higher than pure drugs [19]. Also, citric acid is used as an acidifier for the microenvironment to liberate H^+^ (decrease pH) and enhance the solubility of model drugs (posaconazole), and it produces co-amorphous powder with a higher dissolution, AUC, and C_max_ than crystalline drugs. Moreover, it has good stability because of low recrystallization moisture absorption of this formula is very low (3%) [20]. Furthermore, transdermal preparation used citric acid to enhance the permeability of piroxicam with about a 2.3-fold enhancement in flux than the drug suspension [5].

The best molar ratio of carvedilol:malic acid (2:1) was estimated using a phase diagram; in addition, in Tg-molar ratio curve showed the highest Tg at the same molar percentage. This ratio has 1.7 folds higher intrinsic dissolution rate solubility than crystalline drugs. Also, it is stable for 6 months at 40 °C [21]. On the other hand, malic acid formed a co-amorphous powder with atorvastatin that improved solubility and dissolution of atorvastatin by 2.5 and 2.24, respectively, when prepared at a molar concentration of 1:1 [22].

### 3.3. Saccharides

Although co-amorphous saccharide with carvedilol led to lumps formation and exhibited a lower dissolution rate [23], it was widely used in several types of research.

It participated in the transformation of the nucleation-controlled crystallization mechanism to a diffusion-controlled crystallization mechanism that reduced the crystallization of lurasidone hydrochloride by 68.3–361.2 folds at a molar ratio of 1:1, which also had higher physical stability than 2:1 and 3:1 ratios due to charge-assisted hydrogen bonding interactions [24].

Among different preparation techniques, it was found that solvent evaporation had a superior efficacy on the formation of the co-amorphous system between saccharin with olanzapine drug at equimolar concentration, which enhanced solubility by 145-fold. It stayed stable for 24 weeks under 11% and 75% RH [25].

Samipillai et al. also used the same molar concentration to improve the dissolution rate of olanzapine and dasatinib about 21 times and 30 times, respectively, and co-amorphous preparations stayed amorphous for 8 weeks at different storage conditions (40 °C/75% RH) [26].

Formulation of a co-amorphous system is considered a critical step in the preparation of solid dosage form, as production conditions like compression pressure, humidity, and temperature may threaten the stability of co-amorphous powder. So, amorphous olanzapine and co-amorphous powder of olanzapine and saccharide were prepared as tablet dosage form at different compaction pressures and dwell times, and it was found that formulation containing co-amorphous system exhibited faster dissolution rate and superior stability than formulation containing amorphous drug due to it exhibited some crystallization behaviour at low compaction pressure [27]. This was agreed with da Costa et al. who also studied the pressure-induced amorphization technique and it critically affected the bioavailability and efficacy of tablet dosage form due to increasing compaction pressure and dwell time contributed to enhancing amorphization, especially using 155 MPa as compaction pressure and 5 min as dwell time changed structural properties and dissolution profile of resulted tablets [28].

In situ formation techniques were used to prepare saccharide olanzapine co-amorphous as pellets dosage form in one step during the granulation process, which minimizes production cost. Formulation A, which only contained olanzapine and a high water amount at high drying temperature, showed crystallization behaviour; however, it had higher dissolution improvement than formulations that contained a lower water amount. In another country, formulation B, which contained co-amorphous olanzapine, maintains amorphization and has a small enhancement in drug release [29].

Lactose is used as a co-former for diphenhydramine, the homogenous dispersion of components determined by applying of eutectic mixture and estimating of co-melting point (absence of melting peak). Preparations that contained a high concentration of lactose (more than 60%) had better stability for 28 days at 40 °C/75% RH [30].

Modification of carbohydrates by substitution of a hydroxyl group with alkyl or acetyl groups, like acetylated glucose, leads to improving the glass forming ability of naproxen, especially at a molar ratio of 1:5 [31].

### 3.4. Amino Acids

**Figure 2:**
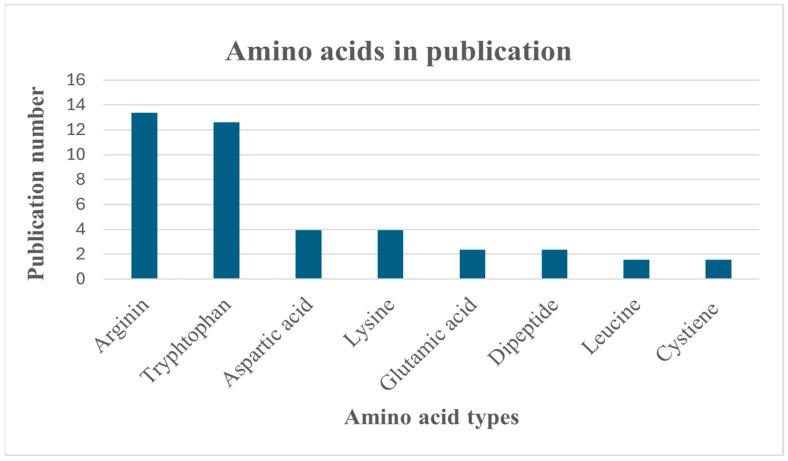
Statistical recording of amino acids in publications.

A lot of numbers of amino acids were currently applied as co-former (Figure 2) as it non-toxic, having high miscibility with other components, high capacity of drug loading [32], and unique structure with different functional groups like carboxyl group that increase the opportunity of molecular interaction with different drugs especially hydrogen bond to obtain the required dissolution and stability [33,34].

#### 3.4.1. Arginine

Arginine is one of the most commonly used amino acids currently applied as a co-former.

It was reported that the effectiveness of co-amorphous indomethacin and arginine at a molar ratio (1:1) in the preparation of different solid dosage forms appeared in the preparation of a multiarticulate system with a superior effect over tryptophan in wet granulation of indomethacin [35].

On the other hand, the arginine and indomethacin co-amorphous system was successfully prepared and loaded on layered pellets using solventless granulation by high high-shear granulator [36].

Also, a tablet dosage form that contains a co-amorphous system of the same content in addition to ibuprofen arginine co-amorphous system was prepared and exhibited high stability when they used mannitol instead of xylitol as diluent [37].

The basic nature of arginine makes it form a stable homogenous mixture with acidic drugs (furosemide and nitrofurantoin) due to salt formation that decreases the hazard of recrystallization, unlike citrulline (neutral amino acid), which has less stability with the same acidic drugs [38]. This was also proved by the formation of the salt in the co-amorphous system between arginine and telmisartan with an acidic character. It enhanced pure drug solubility and dissolution at a molar concentration of 1:2 and stability at a molar concentration of 1:0.5 using the freeze-drying technique [39]. It works by the same mechanism as meloxicam, especially at a molar concentration of 1:3, forming a stable co-amorphous system with high dissolution and drug release [40]. In addition to enhancing the stability of the co-amorphous system with naproxen using freeze-drying [41].

Acid–base interaction was utilized to build a conductimetric titration method that participated in the determination of the most suitable molar concentration of folic acid and arginine (1: 2.5) with the highest solubility (6000 times higher than pure drug), and it had the same results with lysine co-amorphous system [33]. In addition to assisting in the improvement of the dissolution percent of ibuprofen and indomethacin to 74% and 83% respectively, at a molar ratio (1:2), especially at acidic pH = 1.2 [42].

Moreover, kneading of apigenin with arginine forms salt and acts as an alkalizing agent that modifies the dissolution rate and bioavailability of apigenin specifically at a molar ratio (1:2) that is used to prepare disintegrating tablets with acceptable mechanical properties, good content uniformity, and good disintegration time and dissolution percent [43].

Since most of the arginine co-amorphous systems were prepared by ball milling, discrete element method (DEM) stimulation was used as an indicator of the speed of ball collision and it determined the numbers of radicals in a side ball mill that had a proportional relationship with a rotation speed of the ball mill until 600 rpm and consequently co-amorphization and solubility. According to DEM results, arginine-quercetin co-amorphous preparation had a higher number of radicals produced and dissolution percent than the alanine co-amorphous system, and this is due to its ability to ionize the drug, anti-oxidant effect, and it has a guanidino group that stabilizes aromatic components [44]. However, arginine is not always the best choice and it is necessary to add surfactants like sodium lauryl sulphate to co-amorphous glibenclamide to increase dissolution and permeability through parallel artificial membrane permeability assay (PAMPA) by 10 times and 5 times that of amorphous drug powder, respectively [45].

Also, using different isomers (L-arginine or D-arginine) with sodium lauryl sulphate formed a co-amorphous system and modified supersaturation of crystalline hydrochlorothiazide without visual variation of dissolution enhancement of both isomers [46].

Co-amorphous naproxen with arginine system that was prepared by ball milling had a better, faster dissolution rate and enhanced pharmacokinetic properties (C_max_ and AUC_0–24h_) than co-crystal preparation formed by liquid-assisted grinding [47].

#### 3.4.2. Lysine

Two-level full factorial design was constructed to optimize the co-amorphization process of indomethacin with lysine using spray drying. The critical process parameters (CPPs) were atomization gas flow (0.5 and 1.4) and outlet temperature (55 and 75 °C), while critical quality attributes (CQAs) were particle size distribution, bulk density, glass transition temperature (TGA), and yield. According to the results, it was found that low atomization gas flow with high outlet temperature ensures complete amorphization of indomethacin by lysine at an equimolar ratio [48]. Moreover, this same co-amorphous system was prepared by ball milling and compared with the co-crystal system that was prepared by the liquid-assisted grinding method, and it showed the highest dissolution percent and stability than the co-crystal system, amorphous system, and crystalline powder [49]. However, the lysine and naproxen co-amorphous preparation required to addition of surfactants that inhibited crystallization and increased the stability period by 18 weeks [41].

Also, lysine proved its effectiveness as a co-former with the basic drug by forming a co-amorphous system with carvedilol, with the highest enhancement of dissolution rate when compared with tryptophan, phenylalanine, and saccharide, although it had not completely amorphized [23]. In addition to the formation of a co-amorphous system with curcumin that had the highest intrinsic dissolution rate (4.7) among other co-amorphous systems of curcumin with other co-former drugs like folic acid, artemisinin, and piperazine [50].

#### 3.4.3. Tryptophan

Tryptophan formed a non-ionic bonding and decreased mobility of indomethacin molecules in the presence of water as a co-solvent using freeze drying and it found that drug loading has a significant effect on dissolution rate that appeared with faster dissolution rate of formulations with ≤70% drug whereas high drug content preparation (≥80%) had a prolonged drug release [32]. Additionally, it forms a stable co-amorphous system with ranolazine at a molar concentration (1:2) using cryo-milling because low temperature leads to diminishing recrystallization [51].

Another study used a racemic mixture of tryptophan (D-tryptophan and L-tryptophan) and it exhibited lower effectiveness as a co-former for indomethacin than D-tryptophan and L-tryptophan due to it having a longer milling time, lower stability (only 3 months), and lower glass transition temperature that were related to more complicated interaction with presence of two amino acid form. This study also proves that using ball milling had a higher glass transition temperature (TGA) and better stability than the spray drying technique due to the co-amorphous system produced by ball milling recrystallizes at higher temperatures than spray drying [52]. Furthermore, studying the effect of compaction force that was applied to form tablets did not find any differences between tryptophan and aspartic acid as co-formers for carvedilol; however, there was a difference in the disintegration time of the produced tablets. Therefore, it is very important to study the possibility of using super disintegrants with non-polar amino acid (tryptophan) due to the longer disintegration time than the aspartic acid co-amorphous system [53].

Applying neutral amino acids like L-tryptophan and L-phenylalanine as co-formers to amorphized flubendazole at a molar concentration of (1:1) did not significantly affect the elevation of the glass transition temperature (Tg) of the drug that was related to weak chemical interaction between the drug and the co-former, as shown in Fourier transform infrared spectroscopy (FTIR) results. Although L-phenylalanine co-amorphous preparation had a higher C_max_ and AUC than tryptophan, it had a lower stability due to transformation in different phases when exposed to moisture [54].

The co-amorphous preparation of tryptophan with spironolactone had superior solubility enhancement and better stability than phenylalanine due to its higher Tg and higher binding energy of hydrogen bond [55].

Also, tryptophan had a superior effect over lysine, aspartic acid, valine, and methionine in improving AUC, inhabitation of griseofulvin precipitation, and without minimizing drug solubility in fasted state simulated intestinal fluid (FaSSIF) medium like other amino acids; moreover, it has a high partition coefficient that makes it suitable co-former for lipophilic drugs [56].

#### 3.4.4. Leucine

Determination of thermal hydration provided additional information about the ability of arginine to form and stiffen hydrogen bonds and enhance the stability of a co-amorphous system containing arginine with indomethacin, more than L-leucine, which had a less strong interaction. On the other hand, L-tert-leucine co-amorphous preparation did not show recrystallization like L-L-leucine and L-norleucine due to local motions of the two isomers led to thermal activation and enhanced recrystallization [57].

Moreover, L-leucine amorphized griseofulvin by hot-melting extrusion. This technique had a beneficial effect in increasing dissolution percent by more than 82-fold that of crystalline griseofulvin due to the use of aqueous acetic acid that participated in the formation of hydrogen bonds within the co-amorphous system [58].

#### 3.4.5. Glutamic Acid

Sometimes, the presence of an extra functional group in the structure of amino acid co-formers leads to improved dissolution of the co-amorphous system that appeared in higher wetting properties and consequently, faster dissolution rate of telmisartan with α-ketoglutaric acid than glutaric acid, and that is related to hydrophilic surface [59].

Furthermore, it produced a complete amorphization and enhancement of intrinsic dissolution rate (IDR) with carvedilol using spray drying method that had a superior effect over ball milling and solvent assisted grinding method [60] and this agreed with another research that mentioned a stable co-amorphous preparation with the same drug at a molar ratio of 1:1.43 [61].

#### 3.4.6. Aspartic Acid

Co-amorphous preparation between aceclofenac and aspartame amino acid at an equimolar concentration, which was prepared as a pellet dosage form, enhanced the rate of the crystalline drug by over 9-fold and showed a higher suppression (61.7%) than the commercial preparation with long stability for 3 months [62]. In addition, it obtained a co-amorphous system with carvedilol at a molar concentration ratio of 1:1.46 and 1:1.5 [53,61].

#### 3.4.7. Cysteine

Although cysteine was not commonly used in co-amorphization, it formed a stable co-amorphous system and improved the dissolution rate of indomethacin at equimolar concentration [63] as well as with meloxicam due to salt formation between the drug and co-former at a molar concentration ratio of 1:2 and 1:3.

#### 3.4.8. Dipeptides

Sometimes, using dipeptide molecules that contain two amino acids as an alternative to individual amino acids, especially non-polar amino acids, produces a co-amorphous system with better physical properties. This appeared through the success of a dipeptide molecule of equimolar arginine and glutamic acid in the amorphization of mebendazole that reduced milling time and extended the stability of co-amorphous preparation for 6 months than the binary system due to a large molecule of dipeptide preventing aggregation and minimizing recrystallization [64].

On the other hand, three different dipeptide co-formers (tryptophan-phenyl alanine, proline-tryptophan, and phenyl alanine-tryptophan) were also used with mebendazole at a molar ratio (1:1), while the binary system of individual amino acids (phenylalanine, aspartic acid, tyrosine, histidine, and glycine) failed in the formation of the co-amorphous system. All of the dipeptide systems had a fast dissolution rate, higher C_max_, and remained stable for three months [65].

The combination that contained methyl ester aspartic acid and phenylalanine at equimolar concentration with different drugs exhibited a higher dissolution effect and physical stability than single amino acids like aspartic acid and phenylalanine. Although the dipeptide system failed to completely amorphize piroxicam, it was successfully used as a co-former with mebendazole and tadalafil at equimolar concentration [66].

### 3.5. Drug–Drug Co-Former

Drug co-formers had beneficial outcomes like synergetic effect, lowering adverse effects, and ensuring better patient compliance; however, the selection of drugs and the molar ratio of the prepared system should be compatible with the therapeutic dose mentioned in clinical studies [67,68]. Different drug–drug co-amorphous preparations that have recently been explored and evaluated, including their dissolution behavior and physical stability (Table 2).

### 3.6. Polyphenols and Alkaloids

Several researchers worked on creating a successful co-amorphous combination between polyphenols and alkaloids. Piperine is an alkaloid that has absorption enhancement properties through inhibition of glucuronide in the intestinal and hepatic system, and this contributed to improving absorption of curcumin besides pharmacokinetic characteristics like C_max_ and AUC_0–24h_ as well as dissolution enhancement at acidic and basic pH by 4.27- and 3.21-fold, respectively [89]. In contrast, the co-amorphous system of piperine with resveratrol had a declined dissolution rate due to gel formation that increased the recrystallization of drugs [90].

Matrine was applied as a co-former to improve the dissolution behavior of tranilast by more than 10-fold above the crystalline form, making it a suitable candidate for the treatment of acute symptomatic cases [91]. Furthermore, co-amorphous powder of matrine group alkaloid specifically sophoridine, matrine, and oxymatrine with resveratrol exhibited a sustained release behavior with approximately 75%, 71%, and 84% within 12 h besides high stability over 9 months at 25 °C/low RH that make it a suitable choice for treatment of COVID 19 [92].

Also, the high miscibility of oxymatrine with florfenicol (ΔpKa = −4.03) enhanced the therapeutic effect of florfenicol as it had an anti-fibrosis effect, anti-bacterial properties, and metabolism regulation and the equimolar co-amorphous system remained stable for 60 days and 30 days at 25 °C/dry conditions and 30 days at 40 °C/75% RH, respectively [93].

The polyphenol moiety of epigallocatechin-3-gallate formed a stable co-amorphous solid with simvastatin and nifedipine at a molar ratio of 3:1, with 4.9 and 3.82 fold higher dissolution than crystalline drugs, respectively, as well as better C_max_ and AUC_0–24h_ [94].

Additionally, curcumin enhanced the efficacy of paclitaxel as it inhibited the efflux of the drug and increased its concentration in cancer cells (higher C_max_), modifying dissolution rate, and physical stability for 6 months at 25 °C/55% RH [95].

### 3.7. Flavonoids

Using flavone natural products like naringin as a co-former had a good impact on the physicochemical properties of drugs as well as provided additional therapeutic effects that showed in enhancing dissolution release percent of pure pyrazinamide by 20-fold and permeability by 3.14-fold at a molar ratio of 1:1; in addition, it has a hepatoprotective effect [96].

Moreover, it suppressed glycoprotein efflux, which improved the permeability and dissolution of sulpiride at molar ratios of 1:1, 1:2, and 1:3. The equimolar concentration had the highest values of permeability and dissolution modification (1.83 and 9.13 fold), while 2:1 exhibited a crystal behavior. On the other hand, the three co-amorphous preparations remained stable for one month at 25 °C [97]. This correlates with the results of Uppala et al., where the same molar concentration (2:1) of fexofenadine and naringin co-amorphous powder had the highest dissolution and permeability improvement by 4 and 5-fold, respectively [98]. Quercetin is another flavonoid that stabilized raloxifene by forming an equimolar co-amorphous preparation with 2.3-fold higher dissolution than a crystalline drug by using of solvent evaporation method, while the ball milling technique and quench cooling failed to form a co-amorphous system [99].

### 3.8. Nucleotides

The acidic nature of adenosine monophosphate and diphosphate makes them a good co-former for mebendazole as well as tadalafil, due to the formation of hydrogen bonds that enhanced dissolution release percent (2.3–2.5 folds for mebendazole and 1.6 folds for tadalafil); moreover, they stabilized both drugs for 9 and 2 months, respectively. In contrast, they did not have a molecular interaction with celecoxib; therefore, they failed to modify its stability [100].

### 3.9. Functionalized Calcium Carbonate (FCC)

FCC is a porous material that is used to modify the dissolution profile of carvedilol as it has a small particle size and large surface area. Among different concentrations of the drug, only 20, 30, and 40% of carvedilol content improved the dissolution release percent by 3 folds than crystalline drugs. Additionally, formulations that contain 30% of carvedilol had remained stable for 19 weeks at 25 °C/dry condition, and that means it had the highest stability of all prepared co-amorphous systems [101].

## 4. Co-Amorphous Inhalation System

Co-amorphization process was usually performed to produce an inhaler preparation with excellent characteristics. It enhanced the fine particle fraction (FPF) of ceftazidime by using leucine, whereas tryptophan did not affect the fine particle fraction (FPF), which makes leucine is better co-former for the inhalable co-amorphous system that contains ceftazidime and roflumilast [1]. This results correlated with another study that estimated the effect of leucine, tryptophan, valine, methionine, and phenylalanine as co-formers for ceftazidime and it was found that leucin had the highest fine particle fraction (FPF) that reached 77%, minimum cohesiveness, higher evaporation duct (ED%), and minimum cohesiveness due to low surface energy while tryptophan has the highest protection of drug from degradation about 51.1% after 10 weeks [102]. Despite tryptophan and leucine not affecting the amorphization of the binary co-amorphous system of theophylline and levofloxacin, they had a beneficial effect on the aerodynamic performance of the same binary system; however, only tryptophan contributed to the modification of the stabilization for one month at 40 °C [98]. Enhancement of fine particle fraction (FPF) was also reported in inhaler co-amorphous preparation between kanamycin and methionine to 18% as well as RF3 µm to 24% while it remained stable at high relative humidity over 53%. (Co-amorphization of kanamycin with amino acids improves aerosolization). Additionally, co-formers can contribute to the therapeutic effect of the drug and this appeared in the co-amorphous system of ciprofloxacin and quercetin that protects the infected epithelial cell in addition to suppressing the pseudomonas aeruginosa factor and reinforcing the effect of the antibiotic. The resulting co-amorphous system had a higher solubility than the crystalline drug by 3–4 fold and it remained amorphous for 6 months at 22–25 °C [103]. Also, a successful co-amorphous inhalation powder between arginine and levofloxacin was prepared with high drug loading (67%) [104].

## 5. Ternary System

The addition of a third component for the drug and co-former system, especially polymer, leads to combining the beneficial properties of individual co-formers.

It is considered a suitable technique for reinforcing the weak molecular interaction of carbamazepine with tryptophan by adding hydroxy propyl methyl cellulose (HPMC) or polyvinyl pyrrolidone (PVP) polymer as a third component. It formed hydrogen bonds with the drug and enhanced dissolution by fourfold that of the crystalline drug, in addition to maintaining the stability of the co-amorphous system for 6 months instead of one week in the amorphous and binary system between the drug and polymers [105].

Another research emphasized the efficacy of adding HPMC to equimolar co-amorphous powder of carvedilol and aspartame that modifies dissolution behaviour and ensures better control of supersaturation besides a higher elevation of glass transition temperature (Tg) than a binary system [106].

Moreover, PVP AV 64 improved solubility and enhanced the intrinsic dissolution rate of the binary system of indomethacin and arginine. Additionally, using ball milling ensures complete amorphization; it did not have a significant effect on the dissolution rate, unlike hot melt extrusion, which had better drug distribution and faster dissolution rate than pure drug and binary systems [107].

Excipients like mannitol or sodium lauryl sulphate were also employed as a third component in the equimolar concentration of spray-dried formulation that contained tadalafil with alanine. It had 6 and 2 times higher release percent than crystalline drug and binary system of drug with amino acid, respectively, in addition to enhancing the onset of erection by 2 times due to enhancement of pharmacokinetic parameters (C_max_, AUC_0-∞_ values, and T_max_) [108].

This also happened at the addition of cinnamic acid or hydroxycinnamic acid or ferulic acid to the binary system of andrographolide and oxymatrine at an equimolar concentration that extends of paralysis time of nematodes and suppression of Alzheimer’s disease activity due to increasing pKa value, decreasing interaction parameter(Emix) and decreasing Flory–Huggins interaction parameter (χ,) that indicated the presence of intermolecular interaction and higher miscibility within the co-amorphous system. On the other hand, the three excipients formed a stable co-amorphous system with andrographolide and oxymatrine for 18 months and all of them enhanced solubility, dissolution, and intrinsic dissolution rate of the crystalline drug, especially cinnamic acid by 2.3, 3.68, and 11.5 folds, respectively [109].

Taurocholic acid sodium as a surfactant is applied to modify the dissolution rate of a binary system of gliclazide-triamterene [73]. Despite a ternary co-amorphous system that includes α-lactose monohydrate and microcrystalline cellulose having better dissolution properties than the binary system of glipizide and valsartan, it had lower stability than binary systems [72].

## 6. Co-Former Selection

Although the co-former selection is a critical step that strongly affects the formulation of a co-amorphous system, sometimes, there are no specific selection criteria (Table 3) other than a screening method that depends only on trial and error and is applied on amorphization of carvedilol, mebendazole, carbamazepine, furosemide, indomethacin, and simvastatin with phenylalanine, arginine, glutamine, lysine, leucine, isoleucine, threonine, cysteine, tryptophan, aspartic acid, methionine, valine, tyrosine, glutamic acid, proline, serine, asparagine, glycine, alanine, and histatin and it showed that non-polar amino acids like tryptophan and phenylalanine were the best choice for all model drugs while acidic drugs like indomethacin and furosemide form a stable co-amorphous system with basic amino acids like arginine and lysine due to salt formation and it had a pK_a_ difference more than 3 [110] and this was also reported in another screening study that was carried out on 11 amino acids as co-formers for the same six model drugs that were mentioned in the previous study. The amino acids were classified into three groups that include the following: polar (cysteine), basic (arginine, histidine, and lysine), and non-polar (leucine, isoleucine, methionine, phenylalanine), and then, it was found that salt formation between acidic drug and basic amino acid lead to form a stable co-amorphous system with high glass transition temperature and faster solubility especially combination of basic amino acid with indomethacin and furosemide, while cyclic non-polar amino acid had a good co-formability properties with neutral and basic drugs. In contrast, the aliphatic non-polar amino acids like valine, methionine, isoleucine, and leucine did not form a complete amorphous preparation [111].

Amino acids specifically arginine exhibited a superior effect with furosemide at the molar concentration (1:2) over other P-glycoprotein inhibitors like quercetin, piperine, and verapamil hydrochloride in the improvement of dissolution behavior by 3 folds in addition to drug permeability by 9.5 folds; however, it forms a sticky texture as high relative humidity (60%) because it had hygroscopic properties [112].

In other research, arginine co-former was excluded despite it having a higher glass transition temperature (Tg) and it was substituted with tryptophan and phenylalanine because of their higher stability than the arginine-furosemide co-amorphous system, in addition to their higher ability to form a co-amorphous system with furosemide, and the best molar ratios were 0.527 for phenylalanine and 0.423 for tryptophan, which was determined based on eutectic point and heat of fusion [113].

### 6.1. Modern Computational Techniques

Recently, there have been a lot of useful techniques that contributed to the selection of suitable co-formers, including for the co-amorphization process, like the following:

#### 6.1.1. Molecular Descriptor and Partial Least Squares Regression (PLS)

The molecular descriptor is an essential tool used in the prediction of physicochemical characteristics of co-amorphous systems, besides the determination of molecular interaction through the calculation of variables that related to different bonds like weight, rings, Van der Waals molecular volume (VDW_vol), and Van der Waals surface area of the molecules (VDW_area) that determine π–π bond [114].

Moreover, the ability to form ionic bonds and hydrogen bonds was detected using descriptor related to hydrogen bonding and molecular interactions (h_ema), the number of hydrogen bond acceptors (a_acc), the number of hydrogen bond donors (a_don), and the acidity constant (pKa) while Hansen solubility parameter (D d) value estimated drug and co-former similiters and increasing of rings numbers with low D d ensure the formation of π–π bond that highly contributed in stabilization of co-amorphous system with norfloxacin drug. The results of 17 candidates were analyzed by Pearson correlation coefficient to determine linear correlation and it was found that tryptophan, arginine, phenylalanine, saccharide, indomethacin, and naproxen can be suitable for the amorphization process. Moreover, among those candidates, only tryptophan and indomethacin had a faster dissolution rate and long stability for 6 months [115]. On other hand, molecular descriptor was analyzed by partial least square discriminant analysis (PLS-DA) and this was performed in a screening study for 20 amino acids as a candidate co-former for six drugs that include mebendazole, simvastatin, carbamazepine, furosemide, indomethacin, and carvedilol, and only 39 variables out of 250 descriptors were selected according to variables importance for projection (VIP); these were subjected to the goodness of prediction (53%) and goodness of fitness (61%) analyses, showing the importance of pKa differences in the formulation of the co-amorphous system, in addition to the high accuracy of prediction that appeared through high successful prediction percent of mebendazole co-amorphous system (95%) [115]. This was also applied in another study that analyzed 7 variable descriptors out of 36 variables, and it successfully predicted 81% of the co-amorphous system, besides the highly successful prediction percent of the mebendazole co-amorphous system (90%) [116]. Both researchers distinguished between amorphous and non-amorphous preparations according to their position in the score scatter plot [115,116]. However, Chambers et al. detected variables affected by amorphous and non-amorphous responses based on their position in the loading plot (closeness to amorphous or non-amorphous response), and they found that the logarithm of the partition coefficient and the mixing enthalpy difference (ΔH_mix_) had the highest effect on co-amorphous system formulation [116]. The effectiveness of bislactam as a co-former with 13 drugs resulted in 85% goodness of prediction based on different variables that include mixing enthalpy difference, Hansen parameter, as well as acceptors and donors of hydrogen bond difference. The equimolar concentration of piroxicam, furosemide, mebendazole, and paracetamol failed to form a co-amorphous system with bislactam [117]. Application of a machine learning model by the gradient boosting method is very valuable to predict the most suitable co-formers, as it is based on training data and validation to check accuracy by using a score. It successfully predicted the ability to form a co-amorphous system for 15 out of 19 formulations. It was validated through three different inhalation preparations that included mometasone with bambuterol, mometasone with glycopyrronium bromide, and budesonide and tiotropium with high prediction scores of 1, 0.98, and 0.98, respectively. Also, it was validated experimentally using XRPD, which agreed with model predictions [118].

Hydrophobic interaction between drug and co-former is accompanied by negative ΔH_mix_ and low ΔlogP values to ensure the formation of the co-amorphous system, and this is shown in solubility improvement of crystalline gefitinib by different co-formers like saccharide and sulphathiazole that also enhanced solubility of erlotinib [119].

#### 6.1.2. Density Function Theory (DFT) and Quantum Mechanics

Density function theory (DFT) was applied as a molecular simulation tool for the selection of appropriate co-former according value of binding energy between rifampicin and tromethamine [78], and it emphasized on measure strength of hydrogen bond by total charge transfer, while non-covalent bond was determined by electron density and types of bonds that were distinguished by color in the 3D image that indicates the superiority of cysteine over saccharide for amorphization of lurasidone due to it cationic nature [120].

It was also used as an indicator of the success of the co-amorphous system between curcumin and piperine through the distribution of charges, hydrogen bonds, and binding energy. These results were confirmed by an increase in the intrinsic dissolution rate (IDR) of curcumin and piperine by 15.06 and 6.42 folds, respectively [121].

Quantum mechanics optimization was also used to measure binding energy and it revealed the presence of hydrogen bonds and π–π bonds between ceritinib and rutin and the formed preparation at molar ratio 2:1 had a 40 times higher solubility than crystalline drug in addition to enhancement of C_max_ and AUC by 2.4 and 1.6 times, respectively, and it remained stable for 120 days at 25 °C [122].

#### 6.1.3. Molecular Docking

Molecular docking was utilized to calculate the binding energy of sinopic acid with arginine, histidine, lysine, tryptophan, and proline and it showed the highest binding energy (–1.71 kcal·mol^−1^) with lysine and it increased dissolution and anti-oxidant activity at molar concentration 1:1, whereas higher bonding energy of hydrogen bond and higher glass transition temperature of tryptophan make it better choice as co-former for spironolactone than phenylalanine. Although ball milling produced a co-amorphous system with the highest dissolution rate (12.9-fold) in water and (1.5-fold) in HCL when compared with solvent evaporation and freeze drying, there are no significant differences between the three methods in dissolution and anti-oxidant activity [123]. This computational method was also utilized to select 5 amino acids for practical experiments out of 20 amino acids (arginine, lysine, tryptophan, tyrosine, and glutamine) then only lysine and arginine co-amorphous systems had a good anti-oxidant effect due to their higher enhancement in dissolution rate by 1.5 and 1 folds than crystalline drug, besides the enhancement in permeability by 6 and 5 times than that of crystalline drug [34].

## 7. Process Evaluation

Employment of traditional analytical techniques that include XRPD, differential scanning calorimetry (DSC), and FTIR were essential in all of the mentioned research as XRPD is considered an excellent detective method for the absence of crystal structure and formation of co-amorphous system after preparation and during period of physical stability studies (halo patterns and diffraction peaks). It is usually supported by the results of thermal study using DSC, especially the elevation of Tg of mixed preparation above individual components, which is related to molecular interaction and activation energy and this appeared in higher Tg and lower activation energy of valsartan trimethoprim co-amorphous system at equimolar concentration that related to ionic interaction, in contrast to co-amorphous combination of valsartan and 4,4′-bipyridine at a molar concentration of 2:1 that had lower Tg and higher activation energy due to hydrogen bond interaction [124].

FTIR usually proves the formation of the co-amorphous system by checking the presence of molecular interaction, especially hydrogen bonds between the drug and co-former, which is very important for stabilizing the formed amorphous drugs [124].

Recent research reported the utilization of additional tools providing more visual information about crystal behaviour, like polarized light microscopy (PLM) [37,68,77], in addition to principal component analysis (PCA) and water space technique that simplifies, explains, and confirms FTIR and Raman spectroscopy.

Moreover, solubility and dissolution behaviour were essential evaluated responses to determine the success of the process. It is commonly used in screening studies, especially in comparison between different co-formers as solubility results participated in the exclusion of serine, phenylalanine, isoleucine, tyrosine, and methionine, and selection of histidine and tryptophan, then exclusion of the thermodynamically unstable histidine co-amorphous system. In contrast, the equimolar ofloxacin tryptophan combination exhibited solubility enhancement by about 10-fold than the equilibrium solubility and long stability for 2 months [125].

Additionally, changes in dissolution profile during storage due to recrystallization also play a significant role in the determination of the suitability of arginine, tryptophan, and biotin as co-formers for valsartan, tadalafil, and mebendazole through measuring of intrinsic dissolution rate after 40 days and 80 days. The intrinsic dissolution rate of valsartan tadalafil co-amorphous system diminished after 80 days by 50.9–76.7% and 20.8–76.2%, respectively, while the co-amorphous systems of mebendazole with arginine and tryptophan exhibited phase separation, and the intrinsic dissolution rate was increased by 22.2–50.5% at the same period [126].

### Principal Component Analysis (PCA)

PCA was used as an accessory detector to explain XRPD results of indomethacin and cysteine co-amorphous systems and determine the degree of amorphization and amorphization differences through PC1 and PC2, respectively. Moreover, it proved the importance of the rotation speed of ball milling, which affects friction energy and consequently affects the amorphization process [63].

The second application of PCA in amorphization is explaining FTIR spectra and assisting in the determination of molecular interaction between ceftazidime and tryptophan, as well as carvedilol and tryptophan, and it revealed that PC1 variation in both studies is related to the molar ratio between drug and co-former, with 92% and 93.6%, respectively. On the other hand, PC2 variance for co-amorphous ceftazidime and tryptophan was only 6% and it displayed molecular matrix differences within particles, while the carvedilol and tryptophan system exhibited 5.7% of the variation that related to drug and co-former interactions [102].

Loading score explained positive PC1 mean tryptophan and negative PC1 related to ceftazidime in amorphous form, whereas carvedilol and tryptophan have higher PC1, indicating a high drug amount. Moreover, it determined pyrrole and benzene ring interaction in tryptophan and ceftazidime co-amorphous systems, as well as ether groups of carvedilol interaction with tryptophan; additionally, it estimated the best molar ratio (30:70) as PC2 exhibited a peak at the 30% of carvedilol content with enhanced score values of up to 30%, which indicate a strong interaction between drug and co-former. This is also proved by the highest stability of formulation that contains (30–40%) of carvedilol at room temperature [6,102].

Additionally, it checked structure variations between carvedilol with glutamic acid and carvedilol with aspartic acid by loading plot, besides similarities of different molar concentrations according to clustering in the score plot [61].

Score plot shifting is also an indicator of the increasing amount of lactose co-former used in the amorphization of diphenhydramine [30].

PCA contributed to the differentiation between different Raman spectra of samples during the dissolution test and estimated physiochemical properties changes of samples with time in the case of formulation of co-amorphous systems from naproxen and naproxen sodium. On the other hand, it was used to explain spectral results of low-frequency Raman spectroscopy (LFR) and consequently it determined the effect of different storage conditions on the crystallization of kanamycin through the classification of co-amorphous preparations of different co-formers [127].

## 8. Discussion

This review presents an in-depth summarization of extensively used co-formers in the last decade, including strengths and limitations.

There are many solubilization techniques that are currently used in pharmaceutical formulations, such as salt formation, co-solvent, particle size reduction, solid dispersion, and co-crystallization. All those techniques have many drawbacks, like hygroscopicity, solvent residual (toxicity), lower drug loading, and stability. Therefore, co-amorphization is considered a good alternative technique that ensures better drug loading and good molecular interaction between active pharmaceutical ingredients and co-former, and consequently better stability.

Among different co-formers, amino acid co-formers had the highest utilization in the co-amorphization process due to having two chemical groups that enhance the probability of the formation of hydrogen bonds with different drug structures that stabilize the co-amorphous system; in addition, they are affordable and highly effective in improving dissolution of poorly soluble drugs. Moreover, arginine is predominantly employed due to its acidic nature, which makes it a suitable choice as a co-former for basic drugs, while tryptophan is currently applied as a co-former for acidic and basic drugs due to its zwitterion nature and indole ring that interacts with the aromatic moiety of drugs.

Moreover, employing a co-former with a therapeutic effect, either a synthetic drug or natural components like alkaloids, polyphenols, and flavonoids, enforces therapeutic efficacy and minimizes side effects.

Although the ball milling technique is commonly used, especially with amino acids co-formers, it was less efficient than solvent evaporation in some research, and this may be related to it requiring a good optimization of rotation speed and time.

The solvent evaporation method is usually associated with organic acid co-formers, which may be related to ensuring the removal of residual solvent, while spray drying is mainly used to prepare an inhaler co-amorphous system to enhance aerosolization and produce the required particle size range that is effectively deposited in the respiratory system. On the other hand, freeze-drying was rarely used specifically for heat-sensitive drugs, and besides, it works on maintaining the stability of the co-amorphous preparation during the process.

Although the preparation method is a critical variable that influences process quality, a few researchers carried out comparative studies between different co-amorphization methods and they showed a wide variation in over performance of preparation methods according to co-former nature, final dosage form, and chemical structure, in addition to some of those methods like liquid-assisted grinding that can produce co-crystals instead of co-amorphous powder with different physical properties specific process conditions of the drug that makes the selection of preparation method is more complicated and it requires comprehensive literature about co-amorphization of different drugs.

Application of the co-amorphization process is still a promising technique that is not commercially applicable due to a lack of information about the solubility enhancement mechanism and long-term stability to prevent drug recrystallization. On the other hand, scaling up of this process is a critical process that requires re-optimization of process parameters and material attributes in addition to increasing of risk of recrystallization of resulting co-amorphous powder during the manufacturing of solid dosage forms like a tablet, as it contains handling and applied force that may adversely affect the co-amorphous form.

## 9. Conclusions

Preparation of a co-amorphous system with improved drug bioavailability is considered a huge challenge in drug manufacturing, and it requires in-depth knowledge about the co-amorphization process, especially co-formers’ behaviour with different drugs’ nature to obtain a stable co-amorphous preparation with high solubility and rapid dissolution rate using a co-former that is selected based on its physical properties and its ability to form chemical bonds with target drugs.

This systematic review presented the most important parameters that affected the amorphization process, including co-formers that have recently been utilized in co-amorphization processes, such as amino acids, organic acids, and saccharides and it found that amino acids, specifically L-arginine and L-tryptophan, are most utilized co-formers with a wide number of drug and ball milling is the most common preparation method, with some deviation according to the type of co-former. On the other hand, it mentioned additional tools that are used to select co-formers, such as PLS, DFT, and docking techniques, besides PCA, which are applied in the demonstration of FTIR or Raman spectroscopy results and the estimation of the optimum molar ratio of co-amorphous preparation, which can also be determined using a phase diagram.

## 10. Future Perspectives

Although successful co-amorphization technique depends on many variables (molar concentration, co-former types, and preparation methods), most research did not perform process except for the optimization of the final tablet dosage form after amorphization and spray drying method by applying the design of the experiment (DoE) and quality by design (QbD) concept to estimate critical process parameters (CPPs) and critical quality attributes (CQAs) Therefore, the application of (QbD) and risk assessment study during small scaling production to determine suitable design space with the most appropriate variables ranges should be applicable in the pharmaceutical industry to ensure the success of scaling up and production a co-amorphous system with required critical quality attributes (CQAs). Also, further research that emphasizes on comparison between different types of preparation methods to obtain more obvious information that assists in the construction of scientific guidelines for the selection of preparation methods will be required.

## Figures and Tables

**Table 1:** Type of co-formers.

Co-Former	Type
cholic acid, ursodeoxy cholic acid, deoxycholic acid	bile salt
tannic acid, shikimic acid, gallic acid, salicylic acid, 2,3-dihydroxy benzoic acid, 2,4-dihydroxy benzoic acid, citric acid, malic acid, tartaric acid, succinic acid, fumaric acid	organic acids
lactose, acetylated glucose	saccharides
arginine, lysine, tryptophan, leucine, glutamic acid, aspartic acid, cysteine	amino acids
piperine, sophoridine, matrine, oxymatrine	alkaloids
epigallocatechin-3-gallate, curcumin	polyphenols
naringin, quercetin	flavonoids
adenosine monophosphate and diphosphate	nucleotides
functionalized calcium carbonate (FCC)	miscellaneous

**Table 2:** Drug–drug co-amorphous system.

Co-Amorphous System	Preparation Method and Molar Ratio	Dissolution Characteristics	Physical Stability
Atorvastatin-lisinopril [67]	Cryo-milling 1:4,1:2, 1:1, 2:1	1:2 and 1:1 higher than 2:1 and 4:1.	-
Atorvastatin-lisinopril [68]	Cryo-milling 1:4, 1:2, 1:1, 2:1	- 1:2 and 1:1 had a higher by 1.6 times and 2.7 times than amorphous and crystalline atorvastatin. - 2:1 and 4:1 had slow dissolution rate.	1:2 and 1:1 at (40 °C) and had lower moisture absorption than amorphous drugs and dissolution rate did not decrease within 10 days of storage.
Atorvastatin-probucol [69]	Spray drying 1:1	-	1:1 for 35 days at 40 °C/75% RH.
Atorvastatin-isonicotinamide [22]	Spray drying 1:1	Solubility and dissolution of atorvastatin were increased by 2- and 1.94-fold, respectively.	-
Telmisartan-hydrochlorothiazide [70]	Solvent evaporation 1:1, 1:2, 1:3, 2:3	1:3 had 10 times higher than crystalline telmisartan.	1:3 for 180 days at 25 °C, 90 days at 40 °C, and 30 days at 60 °C at 40 °C and 30 days at 60 °C.
Telmisartan-amlodipine [71]	Ball milling 3:1, 2:1, 1:1, 1:2, 1:3	1:1 had a faster dissolution rate.	-
Atenolol-hydrochlorothiazide [7]	Cryogenic milling 2:1, 1:1, 1:2	- Intrinsic dissolution rate of 1:1 is higher than physical mixture and crystalline hydrochlorothiazide by 2.2- and 12.5-fold, respectively. - Molar ratio 1:1 improved C_max_ physical mixture and crystalline hydrochlorothiazide by 1.7 and 7.3, and AUC_(0–24h)_ by 1.4 and 2.6, respectively.	1:1 for 30 days at 4 and 25 °C.
- Gliclazide-valsartan - Gliclazide-valsartan—α-lactose monohydrate - Gliclazide-valsartan-microcrystalline cellulose [72]	- Binary system:ball milling (1:1) - Ternary systems: cryomilling (1:2)	Gliclazide-valsartan-α-lactose monohydrate has a dissolution release percentage higher than 80%.	- Binary systems (1:1) and (1:2) stay amorphous for 4 months at 20 °C/65% RH. - Gliclazide-valsartan-α-lactose monohydrate was recrystallized within 2 months. - Gliclazide-valsartan-microcrystalline cellulose stayed amorphous for 2 months.
- Gliclazide-hydrochlorothiazide - Gliclazide- triamterene [73]	Ball milling 5:1, 1:1, 1:5	- 1:1 no improvement in dissolution rate - Gliclazide-triamterene with taurocholic acid sodium improved the dissolution of triamterene.	1:1 for 6 months at 25 °C/56% RH.
Indomethacin-paracetamol [74]	Quench cooling 2:1, 1:1, 1:2	The intrinsic dissolution rate of 2:1 was increased.	2:1 for 7 and 3 months at 4 and 40 °C.
Indomethacin-paracetamol [75]	Quench cooling 10–90% and 90–10%	-	50:50 for 9 weeks.
Indomethacin-felodipine [76]	Melting method 5:1, 3:1, 1:3, 1:1, 1:5	-	1:1 had the highest stability while 5:1 and 1:5 had the lowest stability at 40 °C.
Indomethacin-apremilast [77]	Quench cooling 1:1	- Solubility increased by 1.64- and 1.85-fold. - Dissolution increased by 14.3- and 49.47-fold. - Drug flux increased by 8.15- and 5.82-fold.	5 months at 40 °C/75% RH.
Rifimpacin-tromethacin [78]	Solvent evaporation 3:1, 2:1, 1:1, 1:2, 1:3	3.5 and 2 folds higher than crystalline form at 30 and 60 min.	2:1 for 180 days at 40 °C/75% RH.
- Gefitinib-bumethanide - Gefitinib-uresemide [79]	- Solvent evaporation - Neat ball milling - Liquid-assisted grinding - Quench cooling (1:1)	2.3 and 3.8 folds higher than crystalline gefitinib.	For 15 months at dry conditions (0 °C and 40 °C) and humid conditions (25 °C/60% RH).
Famotidine-ibuprofen [3]	Cryo-milling 3:7, 1:1, 7:3	-	1:1 for 60 days at 4, 25 and 40 °C.
- Darunavir-ritonavir - Darunavir-indomethacin [80]	Quench cooling 2:1, 1:1, 1:2	higher than crystalline darunavir and lower than amorphous darunavir.	-
- Sinomenine-suphasalazine - Sinomenine-platensimycin [81]	Reduced pressure evaporation	Lower by 20%.	Both were stable for 6 months at 25 °C/75% RH.
- Sinomenine-sulindac - Sinomenine-indomethacin - Sinomenine-naproxen [82]	Solvent evaporation 1:1	The dissolution release percent of sinomenin in all co-amorphous preparations was lower than in crystalline drugs.	All co-amorphous systems had good stability for 4 months at 25 °C/75% RH.
Sinomenine-tranilast [83]	Solvent evaporation 2:1, 1:1, 1:2	- Sinomenin in all co-amorphous preparations exhibited sustained release behaviour. - Tranilast in 1:1 and 1:2 co-amorphous preparations was lower than crystalline tranilast and 2:1.	All saturated with sodium chloride for 6 months at 25 °C/75%RH.
Tranilast-diphenyldramine [84]	Grinding (mortar and pestle) 2:1, 1:1, 1:2	-	For 30 days at 40 °C.
Tolmisartan-ponazural [85]	Solvent evaporation 1:1	Higher than crystalline telmisartan.	The co-amorphous system remained stable for 30 days at 40 °C/75% RH.
Naproxone-naproxone sodium [86]	Ball milling 2:1, 1:1, 1:2	Higher by 2.875-fold over pure naproxone.	1:1 for 2 months at 40 °C.
Curcumin-artemisinin [87]	Solvent evaporation 1:1	Higher solubility and dissolution release than curcumin. The co-amorphous system had two-fold higher bioavailability than the co-crystal system of curcumin-pyrogallol.	-
- Indomethacin-nifedipine - Nifedipine-paracetamol - Paracetamol-celecoxib [88]	Quench cooling different molar ratios	-	- Indomethacin-nifedipine (40% and 60%) for 31–38 days. - Nifedipine-paracetamol (30–50%) for 13–20 days. - Paracetamol-celecoxib (50–50%) for 86–114 days

**Table 3:** Selection criteria of co-former.

Selection Criteria	Properties	Identification Tool
Drug and co-former molecular interaction	Ability to form ionic interaction or hydrogen or π–π bond	Density function theory and molecular docking
Physiochemical and thermal properties	Hansen solubility parameter, molecular weight, LogP, pKa, mixing enthalpy difference (ΔHmix), Van der Waals molecular volume, Van der Waals surface area of the molecules, number of donors and acceptors of hydrogen bond	Molecular descriptor combined with partial least squares regression (PLS)
Pharmacological properties	Compatibility, synergistic effect, and toxicity	Literature review and in silico modeling

## Data Availability

Data are available upon request.
